# The Role of Thrombocyte/Lymphocyte Ratio and Aspartate Transaminase/Alanine Transaminase (De Ritis) Ratio in Prediction of Recurrence and Progression in Non-muscle Invasive Bladder Cancer

**DOI:** 10.7759/cureus.59299

**Published:** 2024-04-29

**Authors:** Mustafa Asım Avcı, Burak Arslan, Oyku Arslan, Enver Özdemir

**Affiliations:** 1 Urology, Republic of Turkey Ministry of Health, Bayburt State Hospital, Bayburt, TUR; 2 Urology, University of Health Sciences Gaziosmanpasa Training and Research Hospital, Istanbul, TUR; 3 Department of Hematology, Basaksehir Cam and Sakura City Hospital, Istanbul, TUR

**Keywords:** recurrence, progression, nmibc, de ritis ratio, bladder cancer, biomarker

## Abstract

Aim: The purpose of the study was to determine the predictive value of platelet-to-lymphocyte ratio (PLR) and Aspartate transaminase (AST)/alanine transaminase (ALT) ratio (De Ritis ratio) for recurrence and progression in non-muscle-invasive bladder cancer (NMIBC).

Methods: A total of 231 patients who underwent transurethral tumor resection between 2016 and 2022 were retrospectively analyzed. Preoperative test results, including AST, ALT, platelet, and lymphocyte counts, were used to calculate the PLR and De Ritis ratio. Univariate and multivariate analyses were performed to identify the predictive factors associated with recurrence and progression.

Results: Based on the ROC curve, 1.19 and 1.21 were identified as the optimal cut-off values of the De Ritis ratio for recurrence and progression, respectively. Furthermore, PLR cut-off values for recurrence and progression were 114 and 118, respectively. There is a significant difference in recurrence-free survival (RFS) and progression-free survival (PFS) between the groups of patients with high and low De Ritis ratios (p = 0.028 and p = 0.021, respectively). In multivariate analysis, De Ritis ratio ≥ 1.19 and European Organization for Research and Treatment of Cancer (EORTC) high recurrence risk were determined to be significant predictors of tumor recurrence. Multivariate analysis also determined that T1 pathological stage, high tumor grade, European Organization for Research and Treatment of Cancer (EORTC) high progression risk, and De Ritis ratio ≥ 1.21 were risk factors for tumor progression.

Conclusion: In our study, the preoperative De Ritis ratio represented an independent predictive factor for recurrence and progression in non-muscle invasive bladder cancer. The use of this biomarker in combination with other diagnostic/predictive tools might help urologists improve the clinical decision-making process in the future.

## Introduction

Urinary bladder cancer is the seventh most commonly diagnosed cancer and is responsible for more than 7% of malignancies in the male population [1]. At initial diagnosis, approximately 75% of patients with bladder cancer have non-muscle-invasive disease, which is defined as a tumor confined to the mucosa (Ta stage, carcinoma in situ) or submucosa (T1 stage) [2]. Patients with non-muscle-invasive bladder cancer (NMIBC) undergoing transurethral resection of bladder tumors may experience recurrence ranging from 30% to 80%, and nearly 20-30% of these may progress to muscle-invasive bladder cancer [3]. In recent years, scientists have developed scoring systems such as the European Organization for Research and Treatment of Cancer (EORTC), Club Urologico Español de Tratamiento Oncologico (CUETO), and the European Association of Urology (EAU) NMIBC 2021 scoring model to predict recurrence and progression.

Currently, there are no readily available, validated diagnostic biomarkers that can predict the recurrence or progression of NMIBC.

Several studies have demonstrated that inflammation can promote all stages of tumorigenesis in cancer development and progression. Inflammation may play an initiating role in cancer development in the presence of an appropriate oncogenic base. It also plays an important role in sustaining carcinogenesis by ensuring cell proliferation, transformation, and angiogenesis through various mediators in the tumor microenvironment [4]. Systemic inflammatory markers, such as the neutrophil-to-lymphocyte ratio (NLR), platelet-to-lymphocyte ratio (PLR), and lymphocyte-to-monocyte ratio (LMR), can be readily calculated from a complete blood count (CBC) and have been reported as useful potential predictors in many types of cancer. While NLR can be used as a biomarker for colorectal, ovarian, and urothelial cancer, PLR can also be used for colorectal and non-small cell lung carcinoma [5-8]. Furthermore, studies have demonstrated that LMR serves as a significant predictor of overall survival for bladder cancer patients undergoing radical cystectomy [9].

Aspartate transaminase (AST) and alanine transaminase (ALT) are enzymes that indicate liver function and damage [10]. The AST/ALT ratio is prevalently used to differentiate between various causes of hepatic disease. It is also referred to as the De Ritis ratio, named after Fernando De Ritis, who performed analysis on aminotransaminases in 1957 [11]. According to the Warburg effect theory, it is known that tumoral activity can increase the AST/ALT ratio [12].

Our current study aimed to determine the predictive value of PLR and AST/ALT (the De Ritis ratio) for recurrence and progression in NMIBC.

## Materials and methods

After receiving approval from the institutional review board, we retrospectively reviewed 245 patients with bladder carcinoma who underwent transurethral bladder tumor resection (TUR-BT) at our department between 2016 and 2022. The data collection process lasted three months and was conducted electronically via computer-based systems. During this period, the demographic and clinical characteristics of the patients were obtained from hospital records and electronic health records. Patients’ characteristics, such as age, gender, Eastern Cooperative Oncology Group (ECOG) score, smoking history, and pathological features, including tumor size, stage, and grade, were all recorded. The patients were followed up according to the Eau Guidelines criteria. Liver disease, chronic inflammatory or hematological disorders, irregular follow-up, or missing data were determined as exclusion criteria. Of 245 patients, 14 were excluded from the study based on these criteria. Finally, informed consent was obtained from all 231 individuals remaining after exclusion.

Routine pre-operative liver function tests and CBC results were used to calculate the PLR and De Ritis ratio. The PLR was calculated by dividing the absolute platelet count by the absolute lymphocyte count.

Patients were classified according to the 2004 WHO/ISUP grading system and staged according to the TNM classification system [13]. The patients were classified according to the EORTC tumor recurrence and progression risk tables. Recurrence scores of 0, 1 to 9, and ≥10 were considered low, intermediate, and high risk for tumor recurrence, respectively. Progression scores of 0, 2 to 6, and ≥7 were considered low, intermediate, and high risk for tumor progression, respectively [14]. Intravesical chemotherapy or immunotherapy was performed based on the patient’s risk scores and the urologist’s preference. The first pathologically confirmed tumor relapse was accepted as a tumor recurrence. In cases where there is an advancement in the T category from carcinoma in situ or Ta to T1 stage (invasion of lamina propria), the emergence of ≥T2 stage, or an increase from low to high grade, it is described as tumor progression [15].

For all statistical studies, SPSS statistical software (version 28.0; SPSS, Inc., Chicago, IL, USA) was utilized. Categorical variables expressed as numbers or percentages were compared with the chi-square or Fisher’s exact test, and continuous variables expressed as the median (range) were compared using the Mann-Whitney U or Kruskal-Wallis test. The predictive cut-off value of the PLR and De Ritis ratio was calculated using the receiver operating characteristic (ROC) curve. Univariate and multivariate Cox regression analyses were performed to identify the independent predictive factors associated with tumor recurrence and progression. The effect of the De Ritis ratio on patients’ recurrence-free survival (RFS) and progression-free survival (PFS) was analyzed using the Kaplan-Meier method. Statistical significance was set at P < 0.05.

## Results

Out of 231 enrolled NMIBC patients in the study, 29 were female and 202 were male. The median age was 62.3 (44-89) years. All NMIBCs were histologically confirmed as “urothelial carcinomas.” The most frequent pathological tumor stage and grade were T1 (58.8%) and high grade (52.3%). A total of 197 patients received intravesical instillation, of which 128 (65%) received BCG instillation. Table 1 displays the baseline characteristics of the patients.

**Table 1:** Baseline characteristics of patients

Characteristics		Patients, n(%)
Age, years, median (range)		62.3 (44-89)
Sex	Female	29 (12.5%)
	Male	202 (87.5%)
Smoking history	Never	67 (29%)
	Former/current	164 (71%)
ECOG score	0	175 (75.7%)
	1	40 (17.3%)
	2	16 (7%)
Tumor size	<3 cm	127 (55%)
	≥3 cm	104 (45%)
Pathological stage	Ta	95 (41.2%)
	T1	136 (58.8%)
Pathological grade	Low	121 (52.3%)
	High	110 (47.7%)
EORTC risk group (recurrence)	Low	49 (21.2%)
	Intermediate	168 (72.7%)
	High	14 (6.1%)
EORTC risk group (progression)	Low	58 (25.1%)
	Intermediate	69 (29.8%)
	High	104 (45.1%)
Intravesical instillation	Absent	34 (14.7%)
	Present	197 (85.3%)

After a median follow-up of 42 (6-69) months, recurrence was detected in 105 patients (45.4%), and tumor progression was detected in 40 patients (17.3%). The median De Ritis ratio of tumor recurrence, non-recurrence, progression, and non-progression groups was 1.30 (1.01-2.86), 1.09 (1.05-2.61), 1.32 (1.04-2.72), and 1.13 (1.0-2.85), respectively.

**Figure 1:**
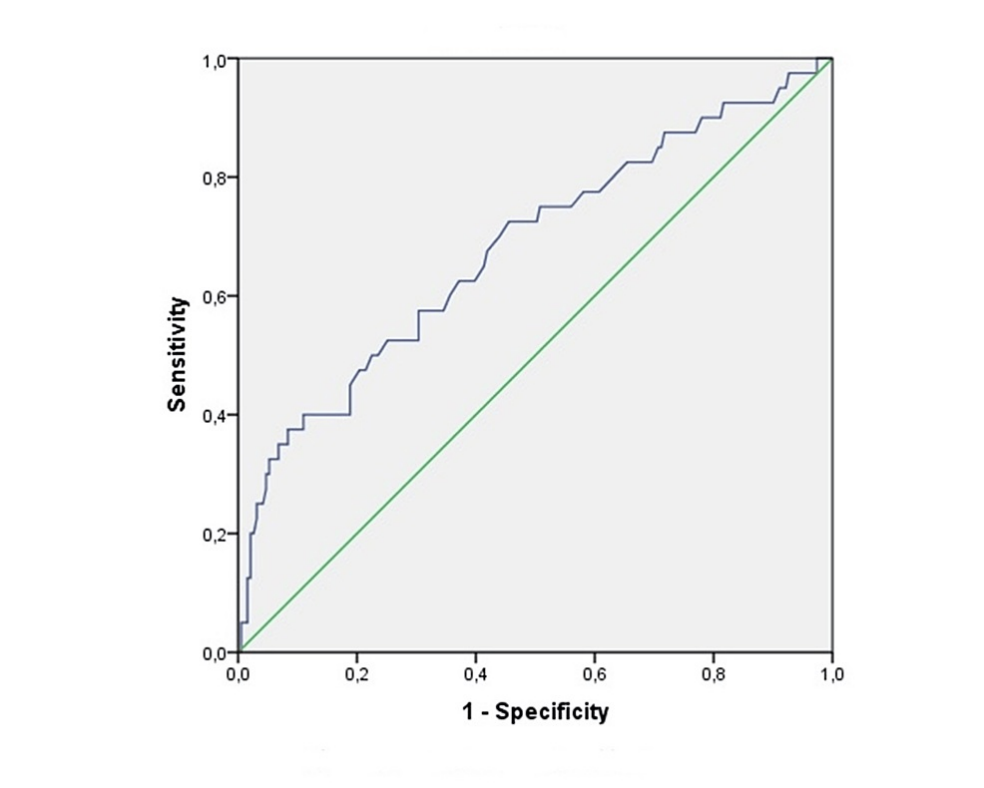
Receiver operating characteristic analysis of De Ritis ratio for recurrence Diagonal segments are produced by ties.

**Figure 2:**
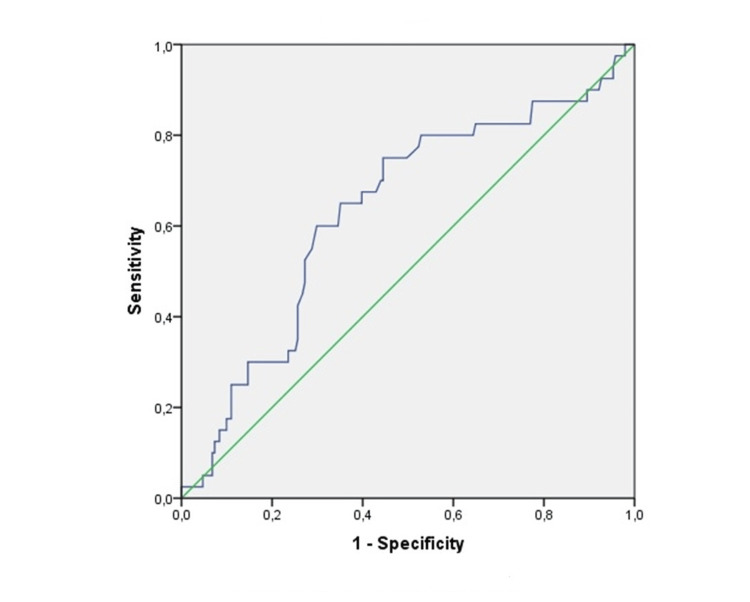
Receiver operating characteristic analysis of De Ritis ratio for progression Diagonal segments are produced by ties.

**Figure 3:**
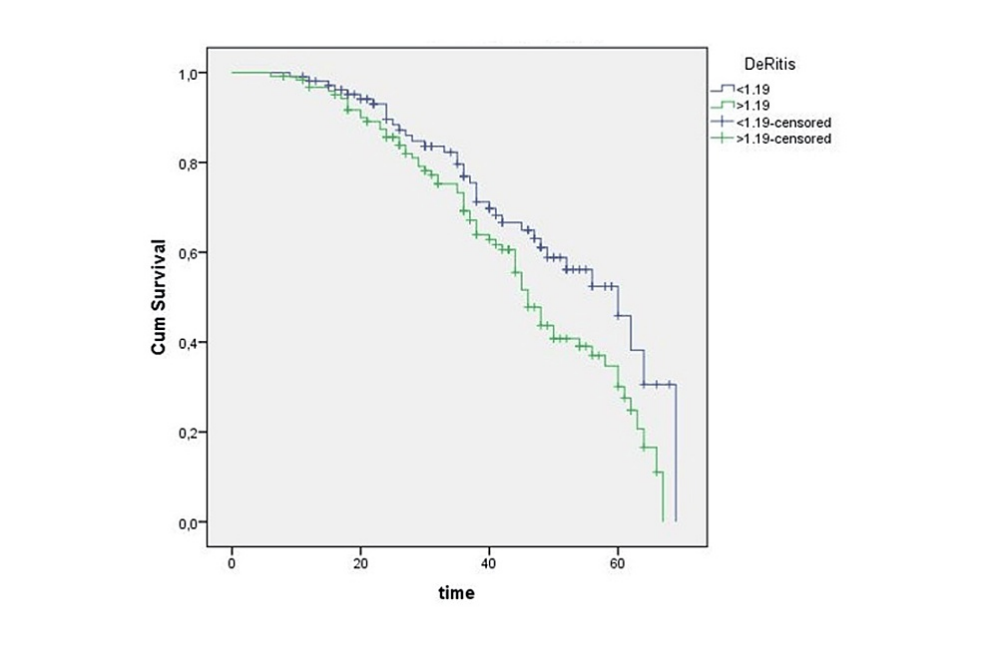
Kaplan-Meier analysis of recurrence-free survival according to the De Ritis ratio Cum. survival: cumulative survival.

**Figure 4:**
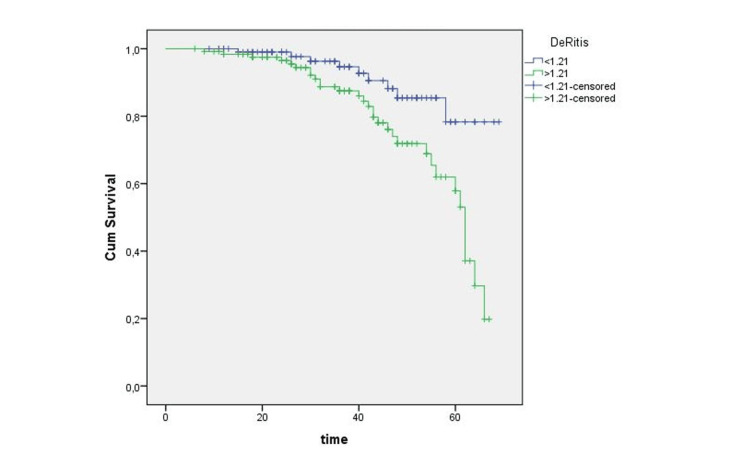
Kaplan-Meier analysis of progression-free survival according to the De Ritis ratio Cum. survival: cumulative survival.

According to the ROC analysis, the optimal cut-off value for recurrence was 1.19, with a sensitivity of 68% and a specificity of 65% (p < 0.05; AUC: 0.672). Similarly, the optimal cut-off value for progression was 1.21, with a sensitivity of 72% and a specificity of 64% (p < 0.05; AUC: 0.680). Furthermore, PLR cut-off values for recurrence and progression were 114 (65% sensitivity, 65% specificity, p < 0.05; AUC:0.635) and 118 (57% sensitivity, 61% specificity, p < 0.05; AUC:0.591), respectively. Figures 1-2 demonstrate the ROC analysis of the De Ritis ratio for recurrence and progression. There is a significant difference in RFS and PFS between the patients with high and low De Ritis ratios (p = 0.028 and p = 0.021, respectively) (Figures 3-4).

Patients with a high De Ritis ratio were more likely smokers (p = 0.028), mostly had T1 stage (p = 0.025), and had high-grade tumors (p = 0.015). On the other hand, high PLR values were significantly correlated with high-grade tumors (p = 0.023). Gender, ECOG score, tumor size, EORTC recurrence, and progression scores were not associated with the De Ritis ratio or PLR values.

In univariate analysis, former or current smoking history, EORTC high recurrence risk, treatment with intravesical instillation, De Ritis ratio ≥ 1.19, and PLR ≥ 114 were found as risk factors for tumor recurrence. However, only De Ritis ratio ≥ 1.19 (OR: 4.31; 1.51-13.2; 95% CI; p = 0.038) and EORTC high recurrence risk (OR: 4.21; 1.49-10.1; 95% CI; p = 0.042) were determined as significant predictors of tumor recurrence in multivariate analysis (Table 2).

**Table 2:** Univariate and multivariate Cox regression analyses for tumor recurrence in NMIBC NMIBC: non-muscle-invasive bladder cancer patients.

		Univariate analysis			Multivariate analysis		
		HR	95% CI	p-value	HR	95% CI	p-value
Age (years)	<65	1(Ref)					
	≥65	1.16	0.80–1.26	0.195			
Sex	Female	1(Ref)					
	Male	1.77	0.49–2.15	0.284			
Smoking history	Never	1(Ref)			1 (Ref)		
	Former/current	5.14	0.88–18.6	0.017	2.11	0.72–2.50	0.118
ECOG score	0–1	1(Ref)					
	2	1.19	0.72–1.61	0.527			
Tumor size	<3 cm	1(Ref)					
	≥3 cm	1.81	0.59–2.11	0.398			
Pathological stage	Ta	1(Ref)					
	T1	2.63	1.40–3.21	0.184			
Pathological grade	Low	1(Ref)					
	High	2.61	1.57–4.45	0.093			
EORTC recurrence risk	Low-intermediate	1(Ref)			1 (Ref)		
	High	4.92	1.67–12.6	0.023	4.21	1.49–10.1	0.042
İntravesical instillation	Absent	1(Ref)			1 (Ref)		
	Present	3.09	1.49–8.65	0.035	2.15	1.03–7.09	0.094
AST/ALT ratio	<1.19	1(Ref)			1 (Ref)		
	≥1.19	4.80	1.62–13.8	0.029	4.31	1.51–13.2	0.038
PLR	<114	1(Ref)			1 (Ref)		
	≥114	2.36	1.12–6.71	0.045	2.23	1.51–6.12	0.088

Univariate analysis also determined that former or current smoking history, T1 pathological stage, high tumor grade, EORTC high progression risk, and De Ritis ratio ≥ 1.21 were risk factors for tumor progression. In multivariate analysis, all of these factors were significant predictors of progression except smoking history (Table 3).

**Table 3:** Univariate and multivariate Cox regression analyses for tumor progression in NMIBC

		Univariate analysis			Multivariate analysis		
		HR	95% CI	p-value	HR	95% CI	p-value
Age (years)	<65	1(Ref)					
	≥65	1.41	0.93–1.81	0.285			
Sex	Female	1(Ref)					
	Male	1.61	0.50–1.85	0.276			
Smoking history	Never	1(Ref)			1 (Ref)		
	Former/current	6.22	1.03–16.1	0.027	3.10	1.65–12.7	0.053
ECOG score	0–1	1(Ref)					
	2	1.46	0.61–1.69	0.410			
Tumor size	<3 cm	1(Ref)					
	≥3 cm	1.60	0.63–2.01	0.572			
Pathological stage	Ta	1(Ref)			1 (Ref)		
	T1	3.82	1.40–8.15	0.031	3.49	1.31–7.79	0.029
Pathological grade	Low	1(Ref)			1 (Ref)		
	High	4.07	1.52–10.0	0.028	4.01	1.31–8.99	0.025
EORTC progression risk	Low-intermediate	1(Ref)			1 (Ref)		
	High	4.97	1.52–12.9	0.019	4.03	1.65–11.0	0.021
Intravesical instillation	Absent	1(Ref)					
	Present	2.83	1.78–5.22	0.081			
AST/ALT ratio	<1.21	1(Ref)			1(Ref)		
	≥1.21	5.19	1.35–15.9	0.015	3.88	1.41–11.1	0.022
PLR	<118	1(Ref)					
	≥118	2.28	1.58–4.71	0.127			

## Discussion

Non-muscle-invasive bladder cancer presents with different rates of recurrence and progression because of the biological heterogeneity of the urothelial tumor and host immune system. Accordingly, the treatment of NMIBC (Ta, T1, carcinoma in situ (Cis)) begins with the transurethral resection of the bladder tumor. Subsequently, an appropriate treatment (intravesical chemotherapy or immunotherapy) is performed based on the patient's individual risk patterns [16]. EORTC and EAU NMIBC 2021 recurrence and progression risk scores are widely used to measure these risks and to classify patients according to the total scores. Otherwise, researchers have been trying to find more useful and reliable serum/urinary biomarkers for years, which provide additional predictive knowledge. As well as several studies mostly focused on the NLR, biomarkers derived from CBC (NLR, LMR, PLR, and platelet-to-hemoglobin ratio), C-reactive protein, and albumin are some of the investigated markers in the literature [17-19].

In hepatocellular damage (e.g., alcohol abuse, hepatitis, and malignancies), AST and ALT enzymes in liver cells are released into the blood, causing an increase in serum AST and ALT levels. Aspartate transaminase (AST) mainly exists in the liver, muscle, kidney, and other tissues, while alanine transaminase (ALT) is predominantly found in the liver [10]. Therefore, ALT specifically indicates liver disease, whereas AST is connected with several diseases affecting other organs [11]. Pathologies causing high proliferation, tissue damage, and increased tumor cell turnover tend to increase AST but not ALT, at least not to the same extent [20]. For that reason, the De Ritis ratio has become a remarkable potential biomarker [20].

Recent study results show that an increased De Ritis ratio is significantly associated with worse clinical outcomes in patients with urological cancers [20-22]. In the study by Laukhtina et al., in which the relationship between oncological outcomes and De Ritis rate in non-invasive bladder cancers was investigated, a significant relationship was found between De Ritis rate and recurrence-free survival, but no relationship with progression was found [23]. According to the meta-analysis by Su et al., a negative correlation was found between a high De Ritis rate and overall survival, tumor-specific survival, and progression-free survival in urothelial cancers [22]. Bezan et al. investigated the relationship between metastasis-free survival and overall survival in patients with localized RCC. They found that an increased De Ritis ratio was an independent prognostic factor associated with poor clinical outcomes in patients with nonmetastatic RCC [20]. Likewise, Gorgel et al. reported a correlation between a preoperative De Ritis ratio greater than 1.3 and a poor prognosis in patients undergoing radical cystectomy [21]. To assess the predictive value of the De Ritis ratio in localized prostate cancer patients, Wang et al. analyzed the relationship between biochemical recurrence (BCR), pathological outcomes, and the De Ritis ratio. Patients with an increased De Ritis ratio were more likely to experience BCR and tended to have worse pathological stages and differentiation than the low-ratio group [24]. Similarly, in our study, we were able to demonstrate that an elevated pre-operative De Ritis ratio was associated with T1 stage, high grade, disease recurrence, and progression.

How can the De Ritis ratio predict recurrence, progression, or survival in bladder cancer patients? Researchers indicated the “Warburg effect” theory in previous studies to answer this question. According to the Warburg effect theory, cancer cells use anaerobic glycolysis to produce adenosine triphosphate (ATP) even under sufficient oxygen conditions [10]. Anaerobic glycolysis produces ATP much faster than oxidative phosphorylation and thus can provide sufficient energy for rapidly proliferating cancer cells [25]. AST plays an essential role in glycolysis by relocating nicotinamide adenine dinucleotide hydrogen (NADH) into mitochondria through the malate-aspartate shuttle pathway [26]. Considering all these, high tumor activity may cause the AST/ALT ratio to increase.

There are several studies on the potential predictive value of PLR in non-muscle-invasive bladder cancers. A meta-analysis by Wang et al., compiling eight studies, showed that increased PLR was associated with worse overall survival in patients with bladder cancer [27]. Zhang et al. assessed the prognostic value of PLR in patients with bladder cancer who underwent radical cystectomy. They found that the pretreatment PLR did not help predict prognosis, but high PLR levels were associated with distant metastases [9]. Kaynar et al. also analyzed the association between PLR and the pathological characteristics of bladder cancer; however, no significant relationship was determined [28]. Although we found a correlation between high PLR levels and a high pathological grade, statistical analysis confirmed that PLR is not a reliable predictor of recurrence or progression.

Bacillus Calmette-Guerin (BCG) instillations are recommended for moderate- or high-risk NMIBC, but the response to this treatment is unpredictable [29]. Numerous biomarkers, including NRL, CRP, and urinary IL-2, have been proposed as valuable predictors of BCG response in patients with NMIBC in the literature [18]. The De Ritis ratio can also be investigated as a potential predictive marker for patients who underwent BCG therapy. Unfortunately, our study cannot assess the predictive value due to the small sample size of patients who underwent BCG instillation.

Our study's limitations include its retrospective design, small sample size, presence of multiple surgeons, and the possibility of undiagnosed inflammatory conditions or liver disease that may affect AST and ALT levels. Furthermore, due to the lack of available mortality data, survival rates could not be calculated.

## Conclusions

Biomarkers obtained from blood tests are cost-effective and easily applicable; they can guide daily clinical practice. The current study demonstrates that the pre-operatively assessed De Ritis ratio might represent an independent predictor of recurrence and progression in NMIBC patients. Its use in combination with other diagnostic/predictive tools (e.g., EORTC, EAU NMBIC 2021 risk tables) might facilitate urologists' clinical decision-making processes in the future.
